# Determination of Thin NiTi Wires’ Mechanical Properties during Phase Transformations

**DOI:** 10.3390/s23031153

**Published:** 2023-01-19

**Authors:** Jonasz Hartwich, Sebastian Sławski, Marek Kciuk, Sławomir Duda

**Affiliations:** 1 Department of Theoretical and Applied Mechanics, Silesian University of Technology, Konarskiego 18A, 44-100 Gliwice, Poland; 2 Department of Mechatronics, Silesian University of Technology, Akademicka 2A, 44-100 Gliwice, Poland

**Keywords:** NiTi, shape memory alloy, actuator, thermographic measurements, phase transformation

## Abstract

The modern industrial and consumer applications in accordance with the concepts of Industry 4.0 and the Internet of Things are characterized by autonomy and self-sufficiency. This has led to an increase in the interest for the so-called smart materials, capable of combining the functionalities of sensors, actuators and, in some applications, control systems. An important group of smart materials are shape-memory alloys, among which nickel–titanium (NiTi) alloys are the most known. In this article, the influence of phase transformation on the mechanical properties of thin NiTi alloy wires was investigated. During the test, the influence of the heating currents on the displacement and the force generated by the thin NiTi wires were analyzed. The temperature of the wires during heating was measured by a thermographic camera. This study proved the maximum value of the wires’ displacement was related to the value of the heating current. During the research, the dependence of the transformation dynamics on the value of the heating currents was also proved. In addition, the influence of the surface inhomogeneity of the thin NiTi alloy wires on the accuracy of the thermographic measurements was analyzed. For the experimental research described in this article, we used the NiTi alloy whose trade name is Flexinol, produced by DYNALLOY (Inc. 2801 McGaw Ave. Irvine, CA, USA).

## 1. Introduction

The sector of smart materials, for many years, was characterized by a dynamic development [1]. This is because smart materials are associated with the most important trends in the industry, such as Industry 4.0 and Internet of Things. Smart materials are defined as man-made or natural materials that respond to appropriate changes in the surrounding environment [2,3] such as pressure, temperature, electric or magnetic fields, hydrostatic pressure or nuclear radiation. There are many smart materials with different reactions to an environmental stimulant; in most of cases, they modify their properties such as size, shape, electrical or magnetic conductivity, optical properties or viscosity.

The increase in the number of smart structures applications has been caused by the increase in the demand of actuators and sensors. The common usage of actuators, sensors and controllers has impacted the device parameters, such as mase, gabbarts and energy consumption. This is particular important for the transport industry because intelligent devices are generally used in cars to develop safe and comfortable communications [4,5,6]. An increase in the weight of vehicles increases their energy demand, which is directly related to an expansion of fuel consumption. This problem enforces the use of maximally light devices. This creates space for the use of intelligent materials which might combine the functionalities of sensors, actuators and controllers. In addition, smart materials may vary the parameters of a car which previously could be changed by using special devices. Examples are dampers or clutches made with magnetorheological fluid [7,8,9]. Applying the latest computer and math solutions improves and accelerates the development of new products based on smart materials. Finite Elements Methods simulations and neural network-based data analyses are just examples of the wide variety engineering and scientific tools [3,10].

An important group among smart materials is that of Shape-Memory Alloys (SMA), which, in response to a change in the environmental conditions, change their internal structure, which leads to a change in the properties of the alloy, such as for example, the shape or electrical resistance. The factor inducing the phase transformation in SMA depends on the type of alloy. Two types of SMA are described in the literature, i.e., thermal shape-memory alloys (TSMA), for which the transformation occurs under the influence of temperature, and magnetic shape-memory alloys (MSMA). for which the transformation is induced by an external magnetic field [11,12].

Among the SMAs, the nickel–titanium alloys have raised great scientific interest and have the largest number of industrial applications [13]. The NiTi alloys belong to the TSMA group. The phase transformation in NiTi alloys is a thermodynamic transformation and involves the transformation of the alloy’s interior structure from martensite to austenite. The transformation between austenite and martensite in NiTi alloys is related to the shape memory effect (SME). The SME is the return of the deformed alloy to the trained shape due to the transformation between martensite and austenite [14,15,16]. In the literature, two types of shape memory effects are described: the one-way shape memory effect (OWSME) [17], and the two-way shape memory effect (TWSME) [18]. The OWSME is the return to the trained shape only during the transformation from martensite to austenite (during heating), which is an inborn feature of SMA, whereas TWSME includes both the austenite–martensite transformation and the reverse transformation (during cooling) [19]. The type of SME for a specific alloy depends on the training process. The training process for TWSME alloys is more expensive than that for OSME alloys, which makes OWSME significantly more commonly used in industrial applications. The phase transformation of NiTi alloys is a high-energy transformation, which means that it can occur even under a large load [20]. This makes shape-memory alloys suitable for use as actuators [21].

There are three main crystal structures in NiTi alloys: twinned martensite, detwinned martensite and austenite; in some commercially produced alloys, a rhombohedral (R) phase may also be observed [22,23]. The crystal structures existing in NiTi alloys are different for their mechanical and physical properties, including their electrical resistance [24]. This makes it possible to identify phase transformations occurring in NiTi alloys, due to the fact that the electrical resistance is an easily measurable [25].

In the literature, the mechanical properties of NiTi alloy wires related to phase transformations are described. O. Tyc et al. [26] conducted displacement-controlled tests on NiTi wires t different temperatures. Displacement tests were conducted until the samples broke. The authors proved that, as the temperature of the sample increased, the amount of unrecovered strain increased. In addition, the authors proved a decrease in the fatigue life of the wire at high temperatures. In their article, L. Fumagalli, et. al. [27] verified the properties of an NiTi alloy wire with a wide range of diameters provided by SmartFlex. The authors analyzed the effect of strain and heating current on the activation time of the wires. In their article, Hunter Song, et al. [28] conducted a validation of the resistance model of NiTi alloy conductors. The authors determined the dependence of wire resistance on the temperature and external load.

The purpose of this paper was to determine the changes in the mechanical properties of a thin NiTi alloy wire correlated with the phase transformations of this alloy. During the experimental research, the dependence of the force generated by the NiTi wire on the heating current and its temperature was measured. The dependence of the sample displacement on the heating current and sample temperature was also determined. The data of the material used for the test along with the process of preparing the test samples are presented in Section 2. The second section also presents the methodology used in the experimental study. The results of the measurements are presented in Section 3. The discussion is reported in Section 4.

## 2. Materials and Methods

In this article, the properties of nickel–titanium alloy wires supplied by Dynalloy (DYNALLOY, Inc., 2801 McGaw Ave. Irvine, CA, USA), whose trade name is Flexinol, were determined. The SMA material used in the research was a low-temperature alloy (symbol LT) whose transformation temperature was 70 °C [29]. The basic properties of the material used according to the manufacturer’s information are presented in Table 1.

In the process of sample preparation, the NiTi alloy wires were cut into pieces, and test bench mounts, that were also electrical contacts, were mounted on their ends according to the method proposed by the manufacturer. The grips on the ends of the test sample could not be attached after the soldering process due to the properties of the alloy surface that made soldering difficult. The attachment method required bending the end of the specimen by about 180°, followed by clamping contact ferrules onto the bent section. After that, the corresponding holder was connected to the ferrules through a soldering process [31]. As test samples, we used wires with two different diameters, i.e., 100 and 150 μm. The desired length of the active SMA wire, that is, without test benches, was 100 mm.

**Figure 1:**
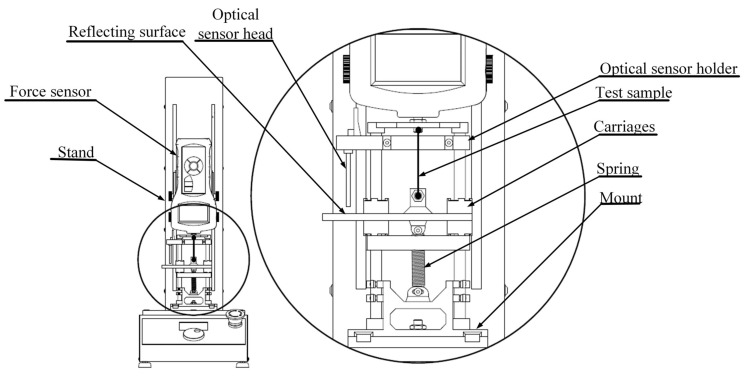
Scheme of the test stand.

The prepared test samples were mounted on a test stand. The samples were attached to the force sensor (connected with the stand) and to the mechanical system that generated a load. A holder with the optical sensor was also attached to the mechanical system. A simplified scheme of the test stand is presented in Figure 1.

The external load-generating assemblage consisted of a carriage moving along two linear shafts, obtained by using a pair of ceramic plain bearings, a holder for the optical sensor, and a mount that allowed the attachment of these components. The carriage provided a mounting location for the tested samples and a mounting location for the reflective surface for the optical sensor. The load-generating element was a spring attached between the carriage and the mount. The dimensions of the used spring were 0.55 mm in section diameter, 5.5 mm in diameter and 35 mm in length. The experimentally determined stiffness of the spring was 0.27 N/mm. A holder for the optical sensor was also attached to the linear shafts in such a way that the sensor head was at a distance of 10 mm from the reflective surface (within the sensor’s measurement range). All the described parts of the mechanical system were made by 3D printing from PLA with the exception of the linear shafts, in aluminum, the ceramic bearings and the spring, in steal.

**Figure 2:**
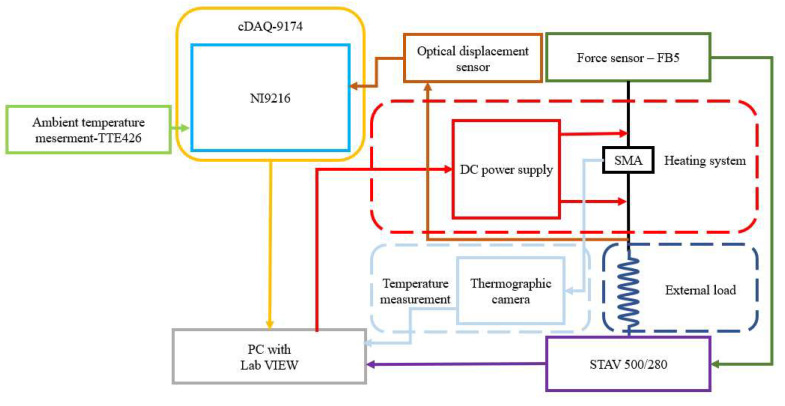
Scheme of the measurement system.

The measurement system consisted of a STAV 500/280 stand (AXIS Sp. z o.o., Gdansk, Poland) for mounting the sample, an FB50 force sensor (AXIS Sp. z o.o., Gdansk, Poland) allowing the measurement of the forces, an RC171 analog optical sensor (PHILTEC, Inc., Annapolis, MD, USA) for measuring the displacements, an RS PRO RS-D3305P DC Power Supply (RS Components Ltd., P.O. Box 5762, Corby, Northamptonshire) which supplied the current flow in the circuit, a Flir A325 thermographic camera with a close-up x1 lens (Teledyne FLIR LLC 27700 SW Parkway Avenue, Wilsonville, OR, USA) for the non-contact measurement of the samples’ temperature, a TTE426 thermocouple for ambient temperature measurements. NI9216 modules (NI, Austin, TX, USA) were used for data acquisition from the optical sensors and the thermocouple. These modules were connected to a cDAQ-9174 (NI, Austin, TX, USA) data-recording system designed to record the measurement data. The measurement system was controlled by an algorithm implemented in the LabVIEW environment. A scheme of the measurement systems is presented in Figure 2.

**Figure 3:**
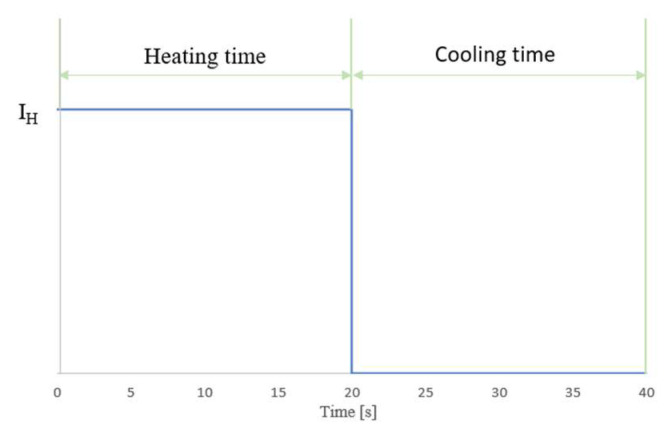
Waveform of the heating current values during the measurement.

In order to measure the strength of a wire made of NiTi alloy, it is necessary to induce a phase transformation in the alloy. This phase transformation for TSMA (which included NiTi) occurred as a result of increasing the temperature of the material by a current according to the Joule–Lenz law. In addition, the phase change of NiTi from martensite to austenite would induce a force-generating change in the system only if this alloy was prestressed. The value of the prestress for a 100 um-diameter sample was 90 MPa, and that for a 150 um-diameter sample was 51 MPa. For these stress values, the deformation of the sample was observable. Therefore, a single measurement was made by moving the stand against the end to which the sample was attached, thus generating a force in the system, while maintaining the stand fixed during the measurement. This was followed by heating the alloy to induce the phase transformation. Heating was accomplished by inducing a current flow in the sample. The measurement lasted 40 s, including 20 s of heating, during which a heating current (I_H_) flowed through the sample, and 20 s of cooling. The waveform of the current in the tested sample during the measurement is presented in Figure 3.

For each diameter of the tested samples, the values of heating current were different and depended on the nominal current specified by the manufacturer. The nominal current for NiTi wires of a given diameter and the measured current values are shown in Table 2.

For each tested sample, measurements were made with different heating currents, starting with the value of the nominal current. Each subsequent measurement was performed using a smaller value of the heating current, until the value for which a significant sample response was no longer observed.

**Figure 4:**
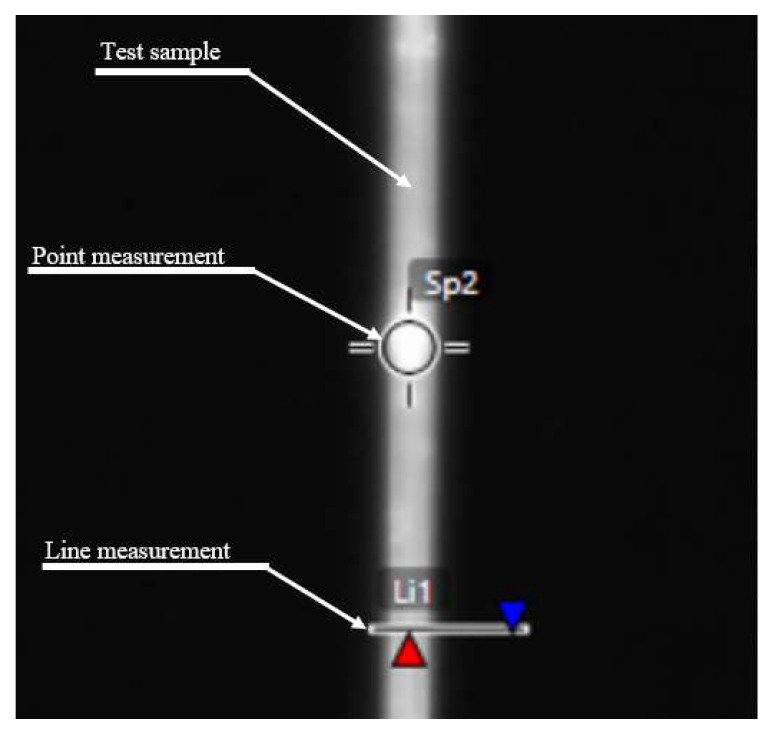
Methodology for the thermographic measurements.

The thermographic camera recordings were analyzed with the dedicated software. The samples’ temperature was measured by point measurements and line measurements. Point measurements read the temperature value at a specific point. Line measurements determine the maximum, average and minimum temperatures along a designated line (as the measurement results, the maximum temperature was considered). The emissivity modulus during the measurements was 0.75. This value was determined experimentally. A theoretical determination of emissivity was difficult because of the surface oxidation of NiTi. Images of the application window are presented in Figure 4. For the line measurements, the maximum temperature value is pointed by the red arrow, and the minimum temperature value by blue arrow.

The measurements were made under stagnant air conditions. Maintaining still air is important due to the susceptibility of thin SMA wires to air movements. Air fluctuations can affect the temperature and stretching of thin NiTi wires (stress-induced twinning), which would cause changes in the observed results.

## 3. Results

**Figure A1:**
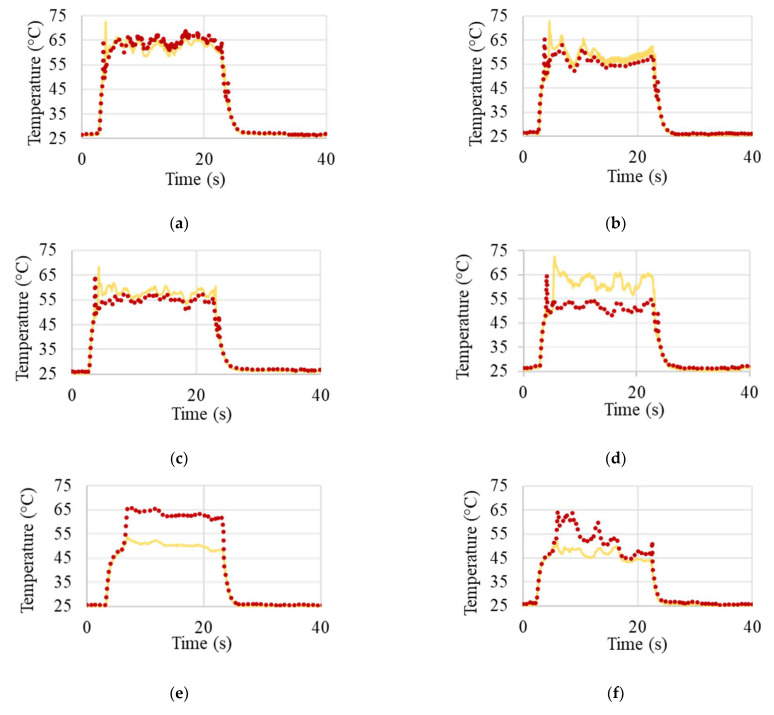

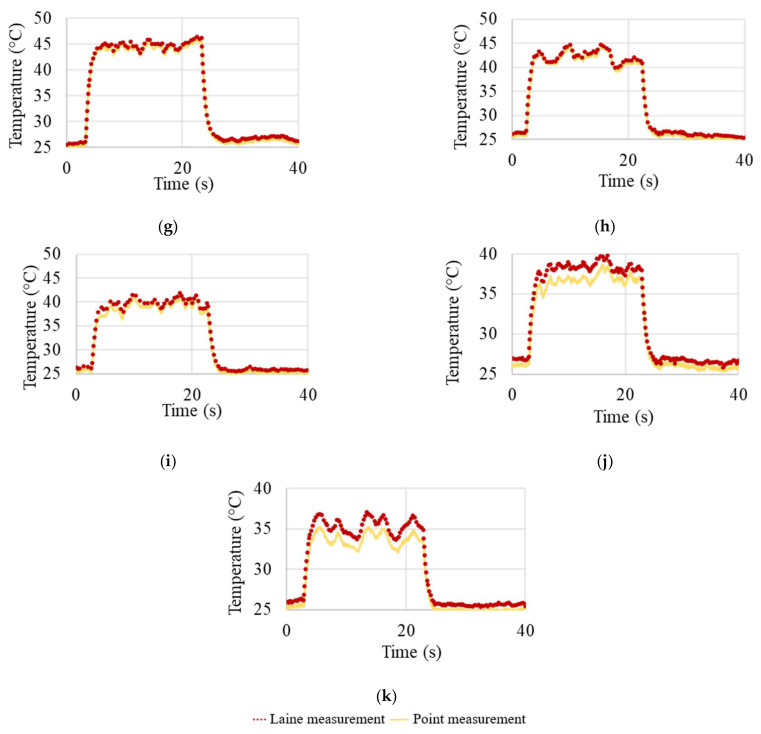
Temperature waveforms of the measurements at different heating currents: (**a**) 200 mA; (**b**) 190 mA; (**c**) 180 mA; (**d**) 170 mA; (**e**) 160 mA; (**f**) 150 mA; (**g**) 140 mA; (**h**) 130 mA; (**i**) 120 mA; (**j**) 110 mA; (**k**) 100 mA. Sample diameter, 100 μm.

**Figure A2:**
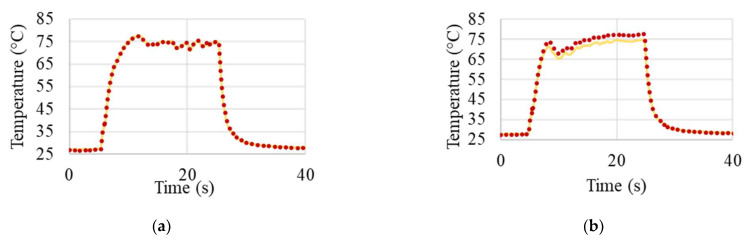

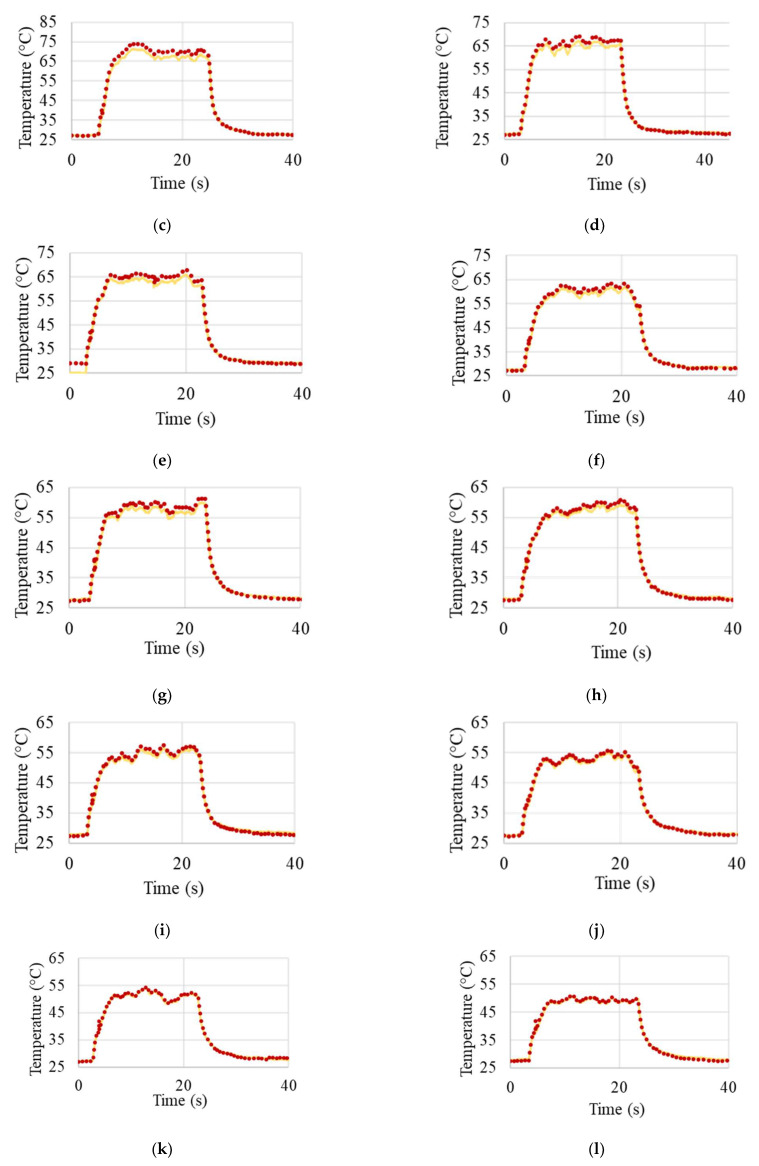

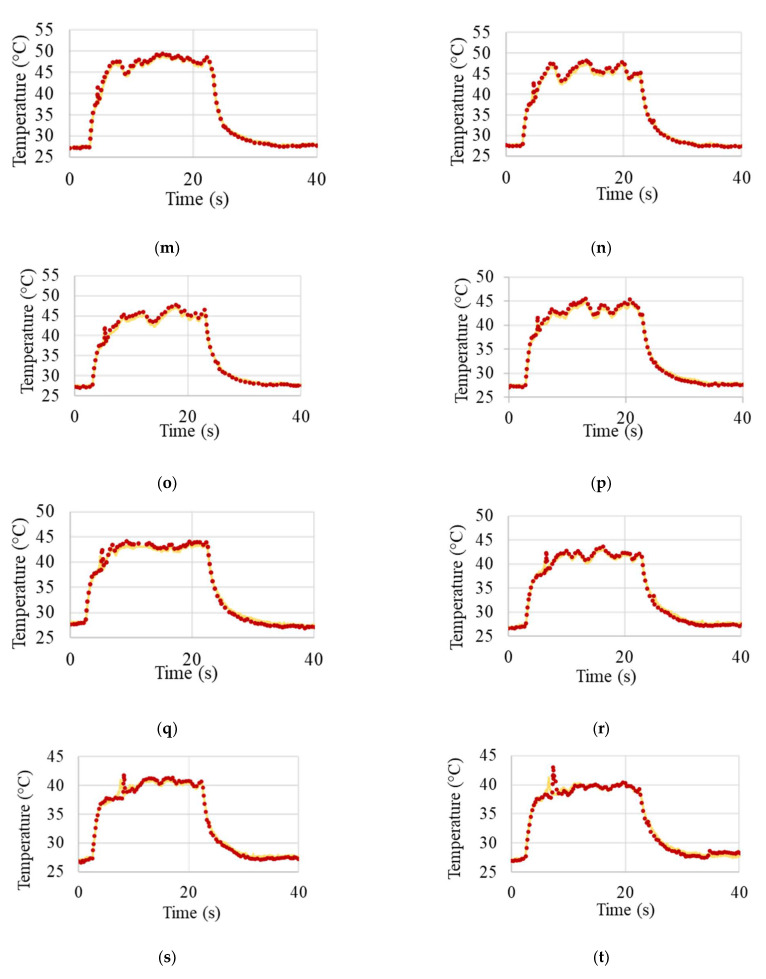

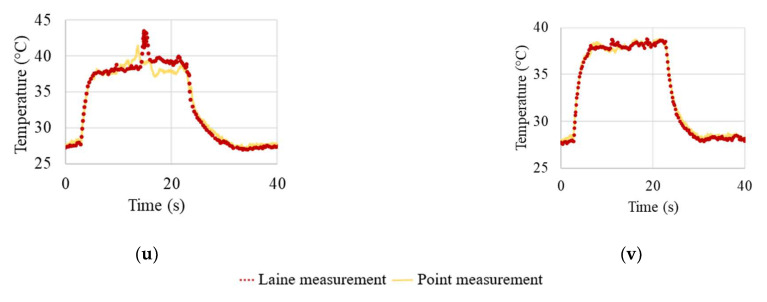
Temperature waveforms of the measurements at different heating currents: (**a**) 410 mA; (**b**) 400 mA; (**c**) 390 mA; (**d**) 380 mA; (**e**) 370 mA; (**f**) 360 mA; (**g**) 350 mA; (**h**) 340 mA; (**i**) 330 mA; (**j**) 320 mA; (**k**) 310 mA; (**l**) 300 mA; (**m**) 290 mA; (**n**) 280 mA; (**o**) 270 mA; (**p**) 260 mA; (**q**) 250 mA; (**r**) 240 mA; (**s**) 230 mA; (**t**) 220 mA; (**u**) 210 mA; (**v**) 200 mA. Sample diameter, 150 μm.

During the tests, displacements and forces resulting from the phase transformation of the thin NiTi alloy wire were measured. In addition, a non-contact measurement of the tested sample’s temperature during the transformation was made. The ambient temperature during the measurements remained in the range from 21 to 22 °C, with small oscillations. This had no significant influence on the measurements. The waveforms of the sample temperature values during the test for the point and line measurements are presented in Figure A1 for the 100 μm- diameter sample and in Figure A2 for the 150 μm-diameter sample. The average temperature value decreased at each measurement, due to the lower value of the heating current. The time for the temperature to rise to the peak value and the time for the temperature to descended changed only slightly at each measurement. For most measurements, the difference in value between line and point measurements was insignificant. However, for some measurements, the difference was significant, and sudden changes in the temperature values were observed.

**Figure 5:**
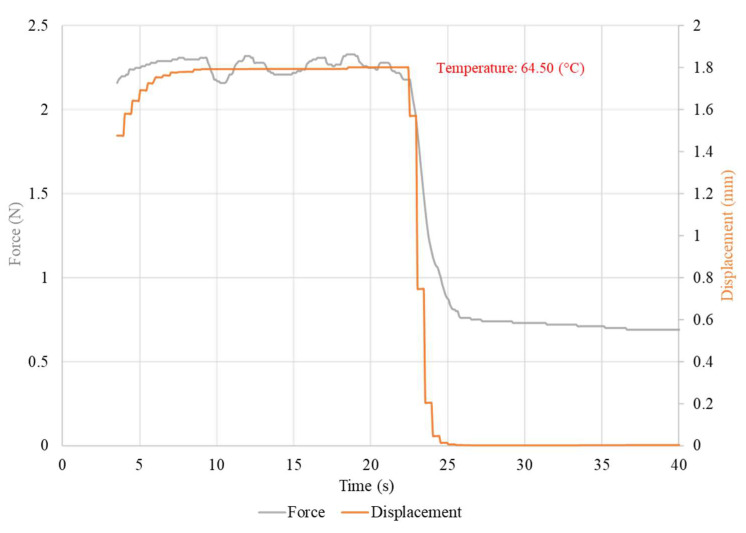
Waveform of the measurement with a 200 mA heating current with the average temperature of the heated sample. Sample diameter, 100 μm.

**Figure 6:**
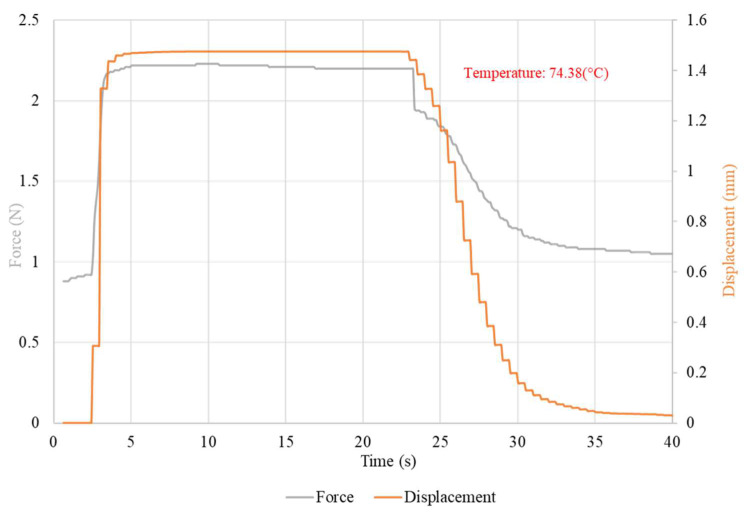
Waveform of the measurement with a 410 mA heating current with the average temperature of the heated sample. Sample diameter, 150 μm.

**Figure A3:**
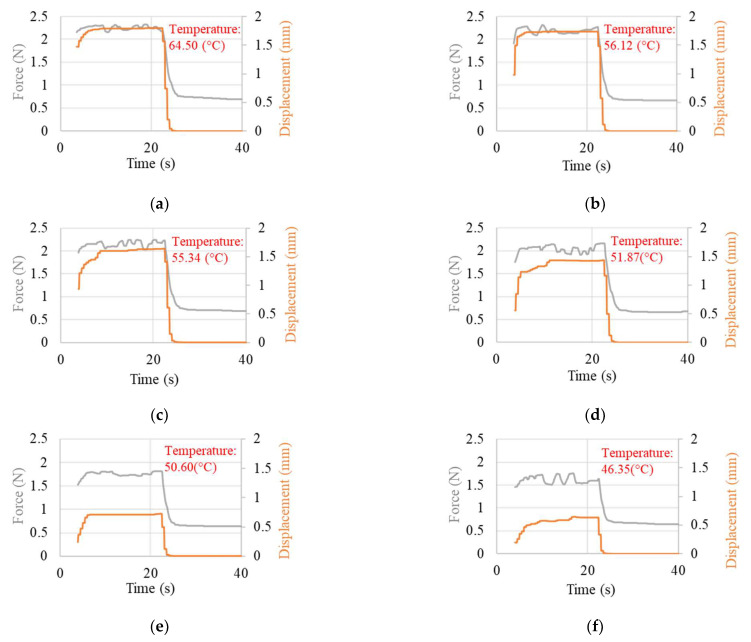

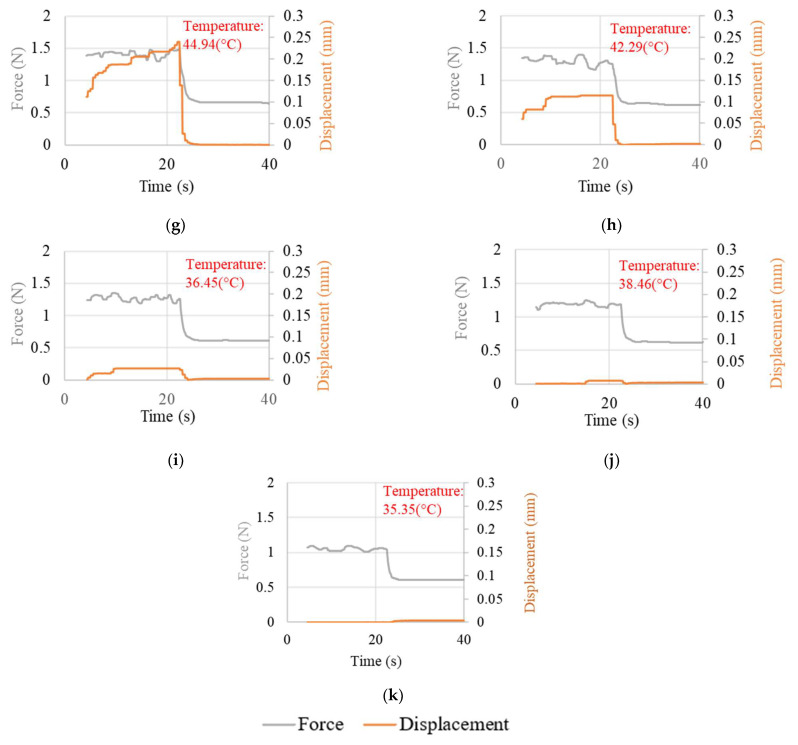
Waveforms of the measurements at different heating currents with the average temperature of the heated sample: (**a**) 200 mA; (**b**) 190 mA; (**c**) 180 mA; (**d**) 170 mA; (**e**) 160 mA; (**f**) 150 mA; (**g**) 140 mA; (**h**) 130 mA; (**i**) 120 mA; (**j**) 110 mA: (**k**) 100 mA. Sample diameter, 100 μm.

**Figure A4:**
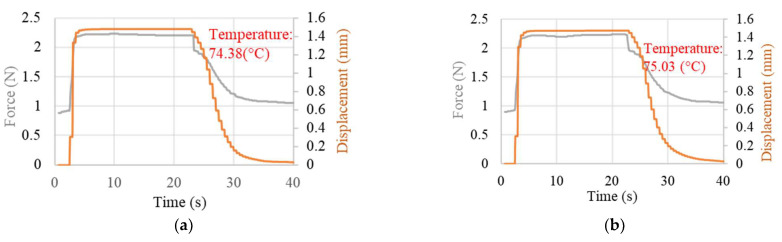

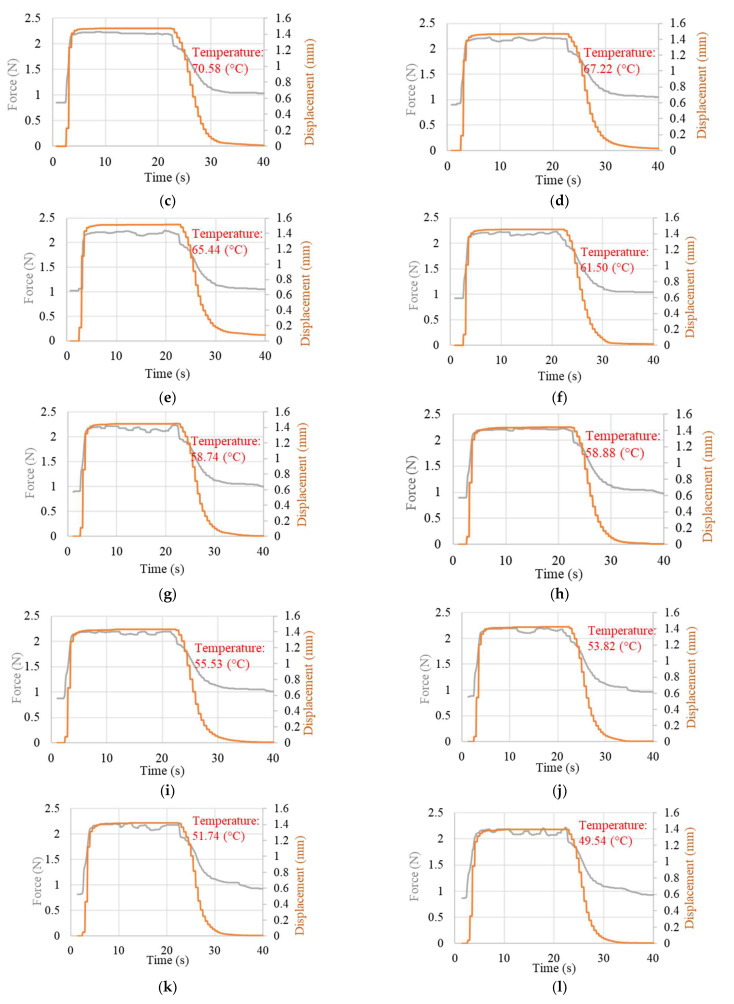

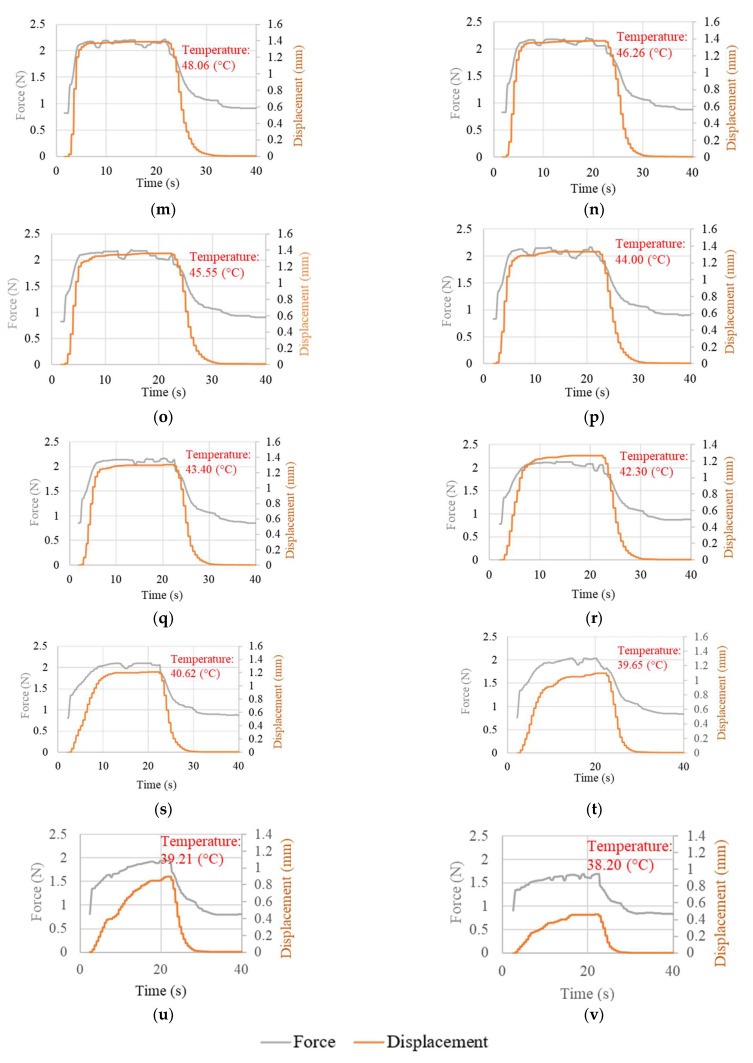
Waveforms of the measurements at different heating currents with the average temperature of the heated sample: (**a**) 410 mA; (**b**) 400 mA; (**c**) 390 mA; (**d**) 380 mA; (**e**) 370 mA; (**f**) 360 mA; (**g**) 350 mA; (**h**) 340 mA; (**i**) 330 mA; (**j**) 320 mA; (**k**) 310 mA; (**l**) 300 mA; (**m**) 290 mA; (**n**) 280 mA; (**o**) 270 mA; (**p**) 260 mA; (**q**) 250 mA; (**r**) 240 mA; (**s**) 230 mA; (**t**)220 mA; (**u**) 210 mA; (**v**)200 mA. Sample diameter, 150 μm.

The waveforms of the displacement value and the force generated by the NiTi wires for both wire diameters, using the nominal value of the heating current, are presented in Figure 5 and Figure 6. The chart also presents the average temperature of the sample after the transformation. The waveforms of the measurements at different values of heating current are presented in Figure A3 for the 100 μm-diameter sample and in Figure A4 for the 150 μm-diameter sample. The average values, as well as the range of these values, of the force and displacement for the tested sample achieved during heating depending on the heating current, for both wires diameters, are presented in Table 3 and Table 4. A relation of the generated force and the achieved displacement to the heating current is observable. As the value of the heating current descended, the values of the generated force and displacement also descended. Initially, the change in these values was for most measurements insignificant and, for measurements at the highest value of the heating current, difficult to observe. The values of force and displacement descended rapidly with heating currents with values close to half of the nominal current for the sample. The value of the sample displacement was stable in most measurements. The exceptions were the measurements at heating currents close to half of the nominal current for the sample. For a sample with a diameter of 150 μm, a high stability of the force value at high heating currents was observed. However, as the value of the heating current decreased, the stability of the force also decreased. This effect was not observed for the 100 μm-diameter sample, for which the generated force was unstable over the entire range of the heating current.

## 4. Discussion

**Figure 7:**
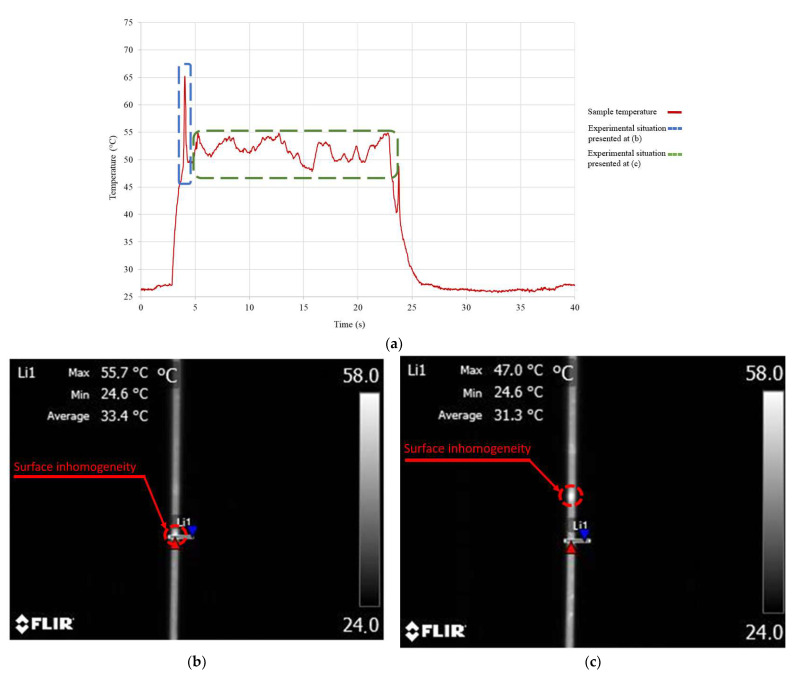
Effect of surface inhomogeneity on the temperature measurements. (**a**) Temperature waveform during the measurement with 170 mA heating current, (**b**) frame from the thermographic camera during the measurements with 170 mA heating current, presenting the inhomogeneity of the surface in the measurement area, (**c**) frame from the thermographic camera during the measurements with 170 mA heating current, presenting the inhomogeneity of the surface outside the measurement area.

During some of the thermographic temperature measurements of NiTi alloy wires during phase transformation, sudden changes in temperature were observed. For some measurements series, significant differences in the measured temperature when using different methods were also observed. This effect was due to the surface inhomogeneity of the tested samples. The inhomogeneity of the samples’ surface was manifested by high differences in the emitted thermal radiation spectrum between surface fragments. During the shrinkage of the tested sample induced by SME, the inhomogeneous areas changed their position relative to the camera lens. In some cases, the inhomogeneous areas moved through the measurement points. This caused sudden temperature changes. This effect is presented in Figure 7.

In some cases, the moved inhomogeneous areas covered the measurements points. For some measurement series, the moved inhomogeneous areas covered the temperature measurement points. In these cases, a significant difference between the measurement methods was observed. The surface inhomogeneity was much greater for the wire with a smaller diameter (100 μm). This was due to its much higher susceptibility to mechanical damage.

**Figure 8:**
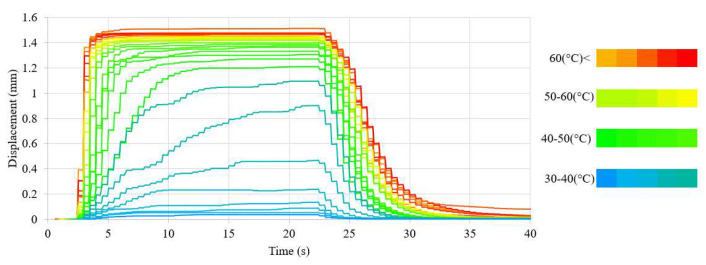
Waveform of the displacement in relation to the average temperature during heating (test sample diameter, 150 μm).

**Figure 9:**
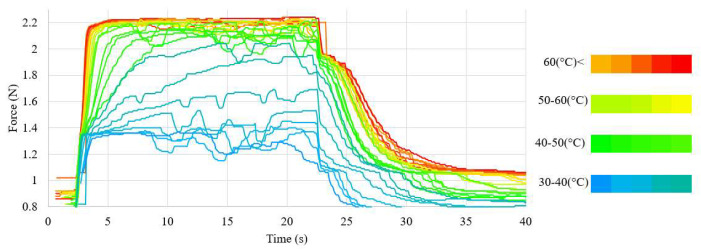
Waveform of the force in relation to the average temperature during heating (test sample, diameter 150 μm).

**Figure 10:**
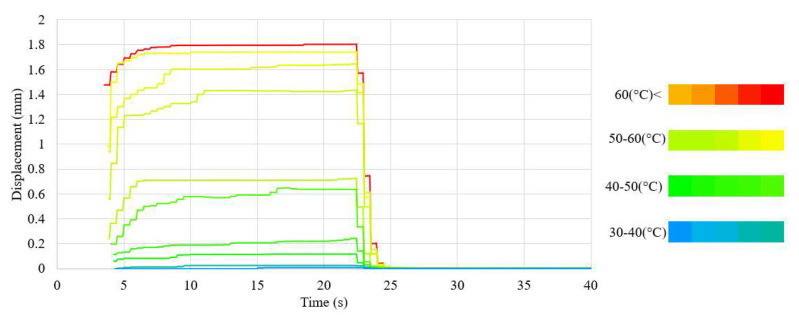
Waveform of the displacement in relation to the average temperature during heating (test sample diameter, 100 μm).

**Figure 11:**
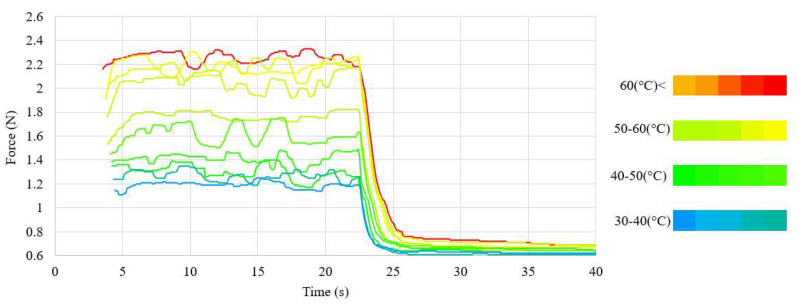
Waveform of the force in relation to the average temperature during heating (test sample diameter, 100 μm).

The average value of the force generated by the sample and the achieved displacement of the sample depended on the heating current. Initially, a decrease in these values occurred in most measurements; the difference between the values obtained with line and point measurements was insignificant. The rate of the reduction increased as the value of the heating current approached half of the value of the nominal current. This was caused by the lower value of the average temperature of the sample for smaller heating currents. The waveforms during heating in relation to the sample temperature are presented in Figure 8 and Figure 9 for the sample with a 150 μm diameter, and in Figure 10 and Figure 11 for the sample with a 100 μm diameter.

**Figure 12:**
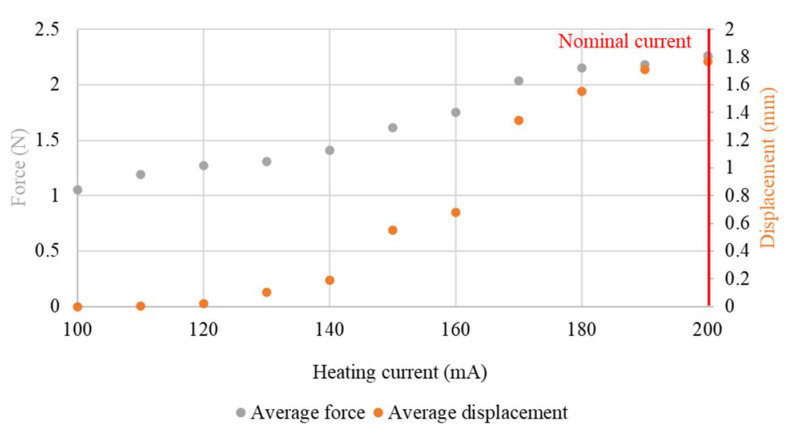
Relationship of average force and average displacement with heating current (test sample diameter, 100 μm).

**Figure 13:**
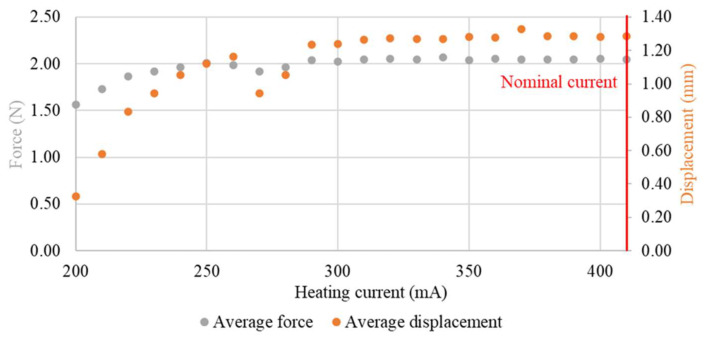
Relationship of average force and average displacement with heating current (test sample diameter, 150 μm).

For lower temperatures, the full phase transformation of the sample did not take place. For this reason, a full return to the trained shape was not obtained. Therefore, in series system with a spring, the value of the generated force would change. The average values of force and displacement during the heating process in relation to the heating current are presented in Figure 12 and Figure 13.

**Figure 14:**
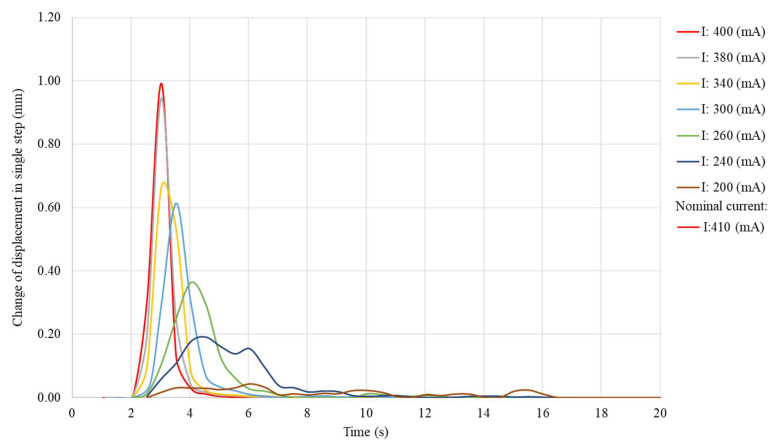
Change of the displacement in a single step of a 150 μm -diameter sample at different heating currents.

A significant change in the values generated by the sample was observable only for a small range of heating currents. Because of incomplete phase transformation, the value of the sample displacement was less stable at lower average temperatures of the sample and its stability decreased with the temperature. This effect was also observable for the generated force. However, for a sample with a diameter of 100 μm, the obtained force values were characterized by noticeable fluctuations over the entire range of the heating current. This effect was probably related to the decrease in activation dynamics at lower values of heating current. A longer activation time increased the influence of external disturbances on the obtained values (in the analyzed setting, disturbances were small fluctuations of ambient temperature and air movements), due to the longer time required for the system to compensate them. The dynamics of activation can be assessed by the change of the displacement during the time in which the system achieves the maximum displacement at a given heating current. The change of the displacement in a single step for a 150 μm-diameter sample at different heating currents is presented in Figure 14.

The sample activation times increased with the decrease of the heating current. The decrease in sample dynamics occurred even despite the fact that, because of incomplete transformation, the maximum sample displacement was small. The dynamics of the transformation remained practically the same for heating currents slightly smaller than the nominal current. The activation time only increased for much smaller heating currents, and the rate of this change decreased as the heating current decreased. For values of heating currents approaching half of the nominal current, activation of the actuator was not observed. In this case, only an oscillation of the displacement value of the sample was observed. The activation times for a 100 μm-diameter sample were not measured, due to the large oscillations of the obtained values. Therefore, it was not possible to objectively determine the end time of the transformation.

## 5. Conclusions

The performed research on thin NiTi alloy wires operating in a series system with a spring illustrated the mechanical properties of NiTi during the transformation and incomplete transformation of martensite to austenite. The dependence of the displacement of the wire and of the generated force on the temperature and the heating current was determined. It was determined that, as the value of the heating current decreased below that of the nominal current, the obtained displacement and the force generated by the wire also decreased. A Significant change in the values was observed only for a very narrow range of heating currents. As the value of the heating current decreased, the transformation dynamics also decreased, which was illustrated by an increase in the activation time of the wires. A decrease in the transformation dynamics involved oscillations in the displacement and the forces generated by the sample. During the measurements, a greater susceptibility of the 100 μm-diameter sample to ambient temperature was also observed. In addition, a significant inhomogeneity of the NiTi alloy wires’ surface was observed during the thermographic measurements. More frequent surface inhomogeneities were observed for the wire with a smaller diameter, due to its greater susceptibility to mechanical damage. The conducted research is a prelude to investigating the use of Dynalloy’s NiTi alloy wires in systems with self-sensing actuators.

## Figures and Tables

**Table 1:** Manufacturer’s stated properties of Flexinol wires [30].

Parameters	Density	Young’s Modulus	Poisson Ratio	Specific Heat	Electrical Resistivity
Units	g/cm^3^	GPa	-	cal/g*°C	μΩ*cm
Martensite	6.45	28	0.33	0.2	80
Austenite	6.45	75	0.33	0.2	100

**Table 2:** Nominal current values and heating current range for a sample of a given diameter [29].

Diameter (μm)	I_N_ (mA)	I_Hmin_ (mA)	I_Hmax_ (mA)	Step (mA)
100	200	100	200	10
150	410	200	410	10

I_N_—nominal current, I_Hmin_—minimal value of the current during the measurements, I_Hmax_—maximum value of the current during the measurements.

**Table 3:** Statistical properties of the measurement data with different heating currents. Sample diameter, 100 μm.

Heating Current (mA)	Average Force (N)	Force Range (N)	Average Displacement (mm)	Displacement Range (mm)
200	2.26	0.17	1.77	0.109
190	2.18	0.22	1.71	0.069
180	2.15	0.21	1.55	0.269
170	2.04	0.25	1.34	0.203
160	1.75	0.15	0.68	0.156
150	1.61	0.25	0.55	0.295
140	1.41	0.19	0.19	0.110
130	1.30	0.23	0.10	0.033
120	1.27	0.16	0.02	0.016
110	1.19	0.11	0.01	0.008
100	1.05	0.08	0.01	0.001

**Table 4:** Statistical properties of the measurement data with different heating currents. Sample diameter, 150 μm.

Heating Current (mA)	Average Force (N)	Force Range (N)	Average Displacement (mm)	Displacement Range (mm)
410	2.05	0.03	1.29	0.006
390	2.05	0.04	1.28	0.004
380	2.05	0.04	1.28	0.009
370	2.05	0.08	1.29	0.017
360	2.05	0.10	1.33	0.021
350	2.05	0.08	1.28	0.015
340	2.04	0.13	1.28	0.019
330	2.07	0.04	1.27	0.035
320	2.05	0.07	1.27	0.029
310	2.06	0.10	1.27	0.027
300	2.04	0.13	1.27	0.040
290	2.02	0.14	1.24	0.049
280	2.04	0.14	1.23	0.039
270	1.96	0.15	1.05	0.071
260	1.92	0.19	0.94	0.113
250	1.99	0.21	1.16	0.106
240	1.96	0.20	1.05	0.156
230	1.92	0.33	0.94	0.126
220	1.86	0.26	0.83	0.185
210	1.73	0.36	0.58	0.110
200	1.56	0.18	0.33	0.123

## Data Availability

Data are contained within the article.

## Appendix A

The waveforms of the temperature during the measurements at different heating currents are presented in Figure A1 and Figure A2.

## Appendix B

The waveform of the measurements at different heating currents are presented in Figure A3 and Figure A4.
